# Safety and tolerability of virucidal hand rubs: a randomized, double-blind, cross-over trial with healthy volunteers

**DOI:** 10.1186/s13756-015-0079-y

**Published:** 2015-10-06

**Authors:** Andreas Conrad, Birgit Grotejohann, Claudia Schmoor, Drago Cosic, Markus Dettenkofer

**Affiliations:** Department of Environmental Health Sciences, Medical Center—University of Freiburg, Freiburg, Germany; Institut für Krankenhaushygiene Oldenburg, Oldenburg, Germany; Clinical Trials Unit, Medical Center—University of Freiburg, Freiburg, Germany; Institut für Umweltmedizin und Krankenhaushygiene, Breisacherstr 115b, D-79106 Freiburg, Germany

**Keywords:** Virucidal hand rub, Transepidermal loss of water, Hand hygiene, Skin tolerability

## Abstract

**Background:**

The hands of the medical staff play an important role in transmission of pathogens in the health care environment. Hand hygiene is efficient, easy to perform and cost-effective. Safety, tolerability and acceptance of hand hygiene preparations play a major role in hand hygiene compliance, and apply, in particular, to formulations with high anti-viral activity.

**Aim:**

Clinical trial to evaluate the safety and tolerability of different virucidal hand rubs.

**Methods:**

In a randomized, double-blind, four-period cross-over trial, healthy volunteers received three different virucidal hand rubs (P1-P3) and a reference product (R) in randomized sequence over a period of 4 days each with a washout period. The primary endpoint was skin barrier function measured by transepidermal water loss (TEWL) after application.

**Results:**

Twenty-two subjects (seven male, 15 female; median age 25, range 21–54) were randomized and started at least one period. TEWL was 22.5; 95 %-confidence interval (CI): 19.6-25.4 after P1, 16.3; 13.5–19.1 after P2, 16.4; 13.4–19.3 after P3, and 24.0; 21.1–27.0 after R; *p* < 0.0001. The percentage of subjects experiencing at least one adverse event (AE) was 86 % with P1, 25 % with P2, 89 % with P3 and 56 % with R. The majority of AEs were skin reactions classified as of mild severity. No serious AEs were observed.

**Conclusions:**

Results were inconsistent. The number of AEs was higher than expected for all products. In summary, there is room for improvement both for hand rub development and the scientific approaches taken to practically and reproducibly evaluate hand rub safety and tolerability.

## Background

The hands of the medical staff play an important role in the transmission of pathogens in the health care environment. In the EU, approximately 4,100,000 patients are estimated to acquire a healthcare-associated infection every year [1]. Hand disinfection with an alcohol-based hand rub is therefore the most important measure to avoid hospital-acquired infections. Not only is it a key parameter for patient safety, but also an important component of the medical staff’s workplace health and safety.

In practice, the hands are not disinfected frequently enough; various investigations have shown that hand sanitizers are only used in every second to third situation where actually necessary [2]. However, only consistent hand hygiene protects patients and staff reliably against transmission of clinical pathogens.

Factors influencing compliance with hand hygiene are manifold and include medical staff workload, occupancy rates, education and knowledge. The accessibility of hand rubs is also an important factor.

Furthermore, the safety, tolerability and acceptance of hand hygiene preparations impacts on compliance. In Germany, hand rub is classified as a drug, approval of which includes evaluation for efficacy, safety and tolerability. Nonetheless, few systematic scientific trials are available on the safety and tolerability of hand rubs. In particular, comparative data are missing for different preparations and their composition. The results obtained in this work on the safety and tolerability of different virucidal hand rubs should provide important information to optimize their safety and tolerability.

With their distinctive environmental stability and easy transmissibility, viruses without capsids like adeno- and norovirus pose a special challenge for the composition of hand rubs [3–5]. Norovirus outbreaks in the health service typically not only lead to illness in patients, but also in staff, often resulting in an acute bottleneck in medical and nursing care and the need for extra nursing staff [6].

For effective hand hygiene against non-enveloped viruses it is recommended to use virucidal hand rubs that either contain special alcohol compositions or are supplemented with phosphoric acid. In practice, healthcare workers often report impaired tolerability and poor compliance with hand hygiene when using virucidal hand rubs. For this reason, virucidal hand rubs appeared to be especially suitable for safety and tolerability testing.

### Methods

#### Study design

In a randomized, double-blind, four-period cross-over trial, healthy volunteers applied three different virucidal hand rubs in four intervention periods: P1 = Ethanol (100 %) 45 g, 1-Propanol (Ph.Eur.) 18 g (Softa-Man® acute, B. Braun Melsungen AG), P2 = Ethanol (99 %) 95 g (Sterillium® virugard, Bode Chemie Hamburg GmbH), P3 = Ethanol (96 %) 57,6 g, 1-Propanol (Ph.Eur.) 10 g (Manorapid® Synergy, Antiseptica GmbH) and a reference product *R* = Ethanol (100 %) 45 g, 1-Propanol (Ph.Eur.) 18 g (Softa-Man® pure, B. Braun Melsungen AG). Volunteers were randomized between the four intervention sequences R/P1/P3/P2, P1/P2/R/P3, P2/P3/P1/R, and P3/R/P2/P1. To simulate regular hand hygiene in practice, the subjects had to carry out repetitive hygienic hand disinfections. Each intervention period lasted 4 days, on which the subjects had to apply a total of 90 mL virucidal hand rub within 90 min (approx. 30 times 3 mL of hand rub). To exclude a carry-over effect, each interventional phase was interrupted by an investigation-free washout period of 10 days.

#### Inclusion and exclusion criteria

Subjects were recruited from students of the Medical Center, University of Freiburg, and nursing students of the Freiburg Academy of Medical Professions. Inclusion criteria were subject’s written informed consent, age > 18 years; legal capacity; healthy in body and mind and not under medical treatment at the time of inclusion. Exclusion criteria were simultaneous participation in other interventional trials or within the last 30 days; known or persistent abuse of medication, drugs or alcohol, relationship of dependence with the sponsor or the investigator, dermatitis of any aetiology (e.g. hand eczema, actinic dermatitis (hands), atopic dermatitis); known allergy to one of the substances under investigation; regular contact with cleaning agents or rubs, or regular immersion of hands in fluid (e.g. nursing care, cleaning company, gastronomy, etc.).

#### Endpoints

The primary endpoint was the skin barrier function measured by TEWL in g/hm^2^ with the Tewameter® TM210 (Company Courage and Khazaka, Cologne).

The following secondary endpoints were assessed: Skin hydration measured by corneometry with the Corneometer CM 820 (Company Courage and Khazaka, Cologne) and skin status clinically evaluated according to the European Society of Contact Dermatitis (ESCD) guideline [7]. The sum of scores 0 = no signs, 0.5, 1, 2, or 3 = marked signs assigned to signs of erythema, roughness/contour, scaling, oedema and fissures was calculated, whereby a sum score ≥3 was regarded as moderate or critical. Satisfaction was assessed by a questionnaire filled out by the subjects. AEs and serious AEs were coded by the Medical Dictionary for Regulatory Activities (MedDRA), Version 13.0.

All the endpoints were assessed at the end of each 4-day application period. TEWL and corneometry measurements were also taken at baseline before each application period. AEs were recorded over the whole study period and assigned to the respective application period if they occurred before application of the next agent.

#### Statistics

Because there was no knowledge of the safety or tolerability of the investigational products, this study was planned as a pilot trial without formal sample size calculation. It was planned to include 20 subjects.

All the analyses were performed in the safety population, including subjects who had started treatment in at least one period. The endpoints TEWL and corneometry were additionally analysed in the per-protocol population (PP), including only subjects having completed all four intervention periods and for whom the endpoints were assessed.

TEWL and corneometry were analysed in linear models including ‘agent’, ‘period’, ‘randomized sequence’ and ‘baseline measurement’ as fixed effects, and ‘subject within sequence’ as random effect. From these models, mean values per agent were estimated with 95 % CI. And the effects of the different agents were estimated as differences between agents with 95 % CI and tested with a two-sided alpha level of 0.05. Additional endpoints were analyzed descriptively.

## Results

### Study subjects

**Fig. 1:**
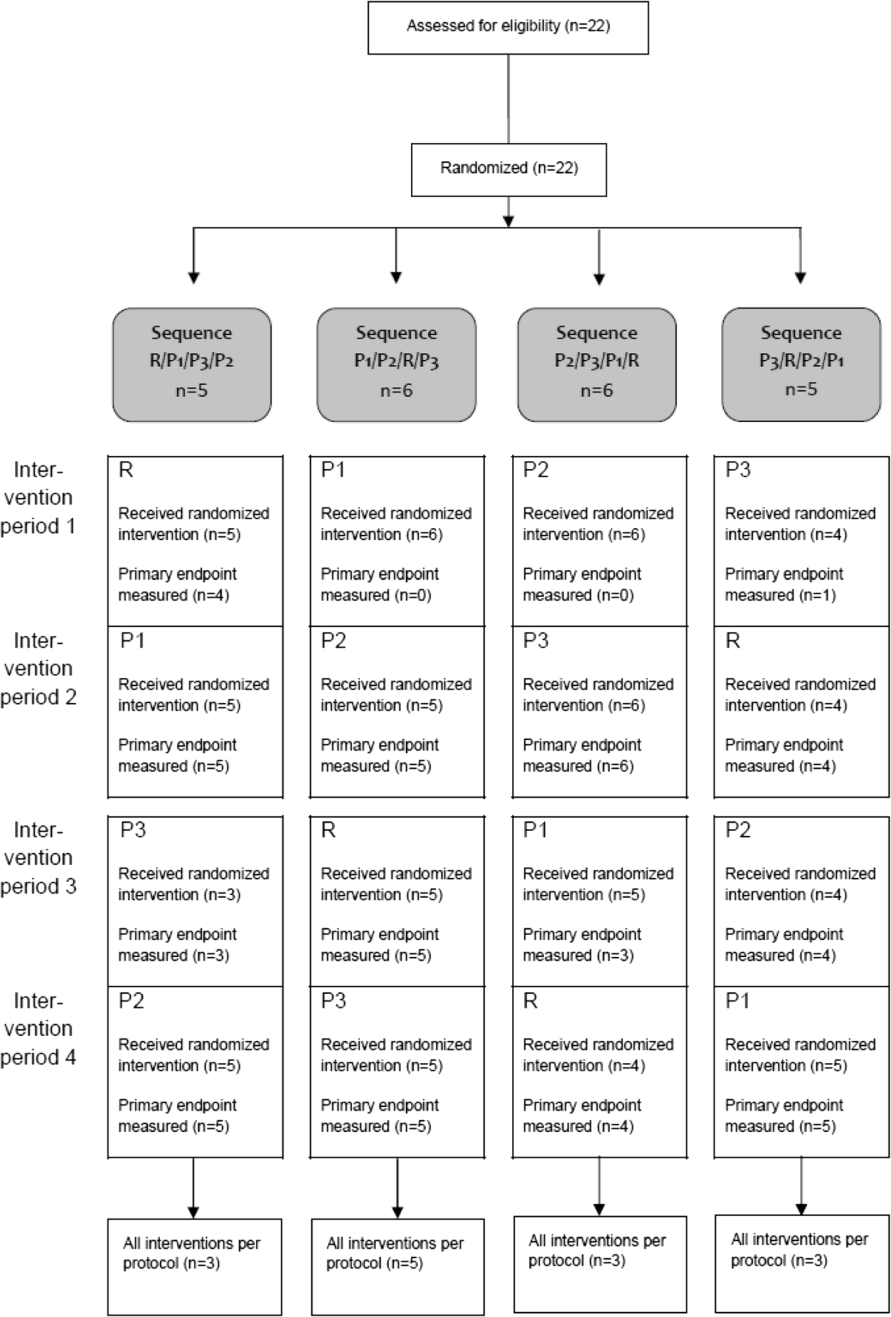
Trial Flow

Twenty-two subjects (seven male, 15 female, median age 25, range 21–54) were randomized. Figure 1 shows the randomized allocation to the different intervention sequences and the flow of the subjects through the four intervention periods in CONSORT diagram. All subjects started at least one intervention period and were thus included in the safety population for at least one agent. The primary endpoint was available for 21 subjects after at least one intervention period. Twenty-one subjects started application of P1 (primary endpoint available for *n* = 18), 20 subjects started P2 (primary endpoint available for *n* = 20), 18 subjects started P3 (primary endpoint available for *n* = 18), and 18 subjects started R (primary endpoint available for *n* = 17). Results are presented for the safety population. In general, results for the PP population (*n* = 14) did not differ from those of the safety population

### Primary endpoint—TEWL

Table 1 shows the results of the post-intervention TEWL measurements by intervention period and agent (mean and standard deviation, SD). The mean results of post-intervention TEWL measurements using the different agents were 22.5; 95 %-CI: 19.6–25.4 after P1, 16.3; 95 %-CI: 13.5–19.1 after P2, 16.4; 95 %-CI: 13.4–19.3 after P3, and 24.0; 95 %-CI 21.1–27.0 after R; test of difference between agents *P* < 0.0001. The differences in TEWL between agents with 95 % CI are given in Table 2. P2 and P3 showed a significantly smaller TEWL than the reference product R, whereas no difference was detected between P1 and R. P2 and P3 also showed a significantly smaller TEWL than P1. No difference was detected between P2 and P3.

**Table 1:** TEWL—by agent and intervention period (safety population, *n* = 22)

Agent												
	P1			P2			P3			R		
Intervention period	n	Mean	SD	n	Mean	SD	n	Mean	SD	n	Mean	SD
1	5	21.26	4.92	6	19.25	6.17	4	13.93	3.39	4	26.93	5.07
2	5	17.92	2.63	5	19.94	9.41	6	15.48	3.08	4	23.98	6.39
3	3	28.03	12.99	4	13.40	2.72	3	20.67	1.33	5	23.74	8.20
4	5	24.08	3.26	5	13.02	2.11	5	17.08	5.09	4	21.50	6.95

P1 = Ethanol (100 %) 45 g, 1-Propanol (Ph.Eur.) 18 g (Softa-Man® acute, B. Braun Melsungen AG), P2 = Ethanol (99 %) 95 g (Sterillium® virugard, Bode Chemie Hamburg GmbH), and, P3 = Ethanol (96 %) 57,6 g, 1-Propanol (Ph.Eur.) 10 g (Manorapid® Synergy, Antiseptica GmbH) , R (reference product) = Ethanol (100 %) 45 g, 1-Propanol (Ph.Eur.) 18 g (Softa-Man® pure, B. Braun Melsungen AG), *n* = number, SD = standard deviation

**Table 2:** Results of TEWL measurements. Differences between agents (safety population, *n* = 22)

Difference to agent									
	P2			P3			R		
Agent	Differ-ence	95 %-CI	P	Difference	95 %-CI	P	Difference	95 %-CI	P
P1	6.16	2.54–9.79	0.0013	6.14	2.38–9.89	0.002	−1.54	−5.33 - 2.26	0.42
P2	-	-	-	−0.03	−3.69 - 3.64	0.99	−7.70	−11.45 - −2.96	0.0001
P3	-	-	-	-	-	-	−7.67	−11.45 - − 3.90	0.0002

P1 = Ethanol (100 %) 45 g, 1-Propanol (Ph.Eur.) 18 g (Softa-Man® acute, B. Braun Melsungen AG), P2 = Ethanol (99 %) 95 g (Sterillium® virugard, Bode Chemie Hamburg GmbH), and, P3 = Ethanol (96 %) 57,6 g, 1-Propanol (Ph.Eur.) 10 g (Manorapid® Synergy, Antiseptica GmbH, R (reference product) = Ethanol (100 %) 45 g, 1-Propanol (Ph.Eur.) 18 g (Softa-Man® pure, B. Braun Melsungen AG)

### Secondary endpoints

The mean results of the post-intervention corneometry measurements were 49.7; 95 %-CI: 40.7–58.6 after P1, 45.4; 95 %-CI: 36.9–53.9 after P2, 64.3; 95 %-CI; 55.3–73.2 after P3, and 48.9; 95 %-CI: 39.6–58.1 after R; test of difference between agents *P* = 0.005. P3 showed a significantly larger corneometry than the reference product R (15.40; 95 %-CI: 4.09–26.70; *P* = 0.0087), whereas no differences were detected between P1 and R or between P2 and R (0.81; 95 %-CI: −10.62–12.23; *P* = 0.89, and −3.50; 95 %-CI: −14.67–7.66; *P* = 0.53, respectively. P3 also showed a significantly larger corneometry than P1 and P2 (14.59; 95 %-CI: 3.53–25.65; *P* = 0.011, and 18.90; 95 %-CI: 8.23–29.57; *P* = 0.0009, respectively. No difference was detected between P1 and P2 (4.31; 95 %-CI: −6.33–14.95; *P* = 0.42).

The skin status score was (palmar, median, range) 0.75 (0–2.0) after P1, 0 (0–1.5) after P2, 1.0 (0–3.0) after P3 and 0.5 (0–1.0) after R and (dorsal, median, range) 0.5 (0–2.0) after P1, 0 (0–3.0) after P2, 1 (0–2.0) after P3, and 0.5 (0–1.5) after R.

Subjective satisfaction after application of the agents is shown in Table 3. The overall impression was positive in 5 % of the subjects after P1, in 60 % after P2, in 17 % after P3 and 0 % after R. In general, P2 showed the best results for all the aspects covered, except smell.

**Table 3:** Results of subject questionnaire—by agent (safety population, *n* = 22)

		Agent							
		P1		P2		P3		R	
Aspect	Assessment	n	%	n	%	n	%	n	%
Smell during application	Pleasant	3	14.3	3	15.0	6	33.3	0	0.0
	Neutral	11	52.4	7	35.0	11	61.1	10	58.8
	Unpleasant	7	33.3	10	50.0	1	5.6	7	41.2
	Missing	1		2		4		5	
Skin feeling after application	Optimal	3	15.0	13	65.0	4	22.2	3	18.8
	Not optimal	17	85.0	7	35.0	14	77.8	13	81.2
	Missing	2		2		4		6	
Sense directly after application	Positive	4	19.0	12	60.0	3	16.7	1	5.9
	Neutral	6	28.6	5	25.0	4	22.2	7	41.2
	Negative	11	52.4	3	15.0	11	61.1	9	52.9
	Missing	1		2		4		5	
Sense while longer application	Positive	1	4.8	11	55.0	1	5.6	1	5.9
	Neutral	4	19.0	6	30.0	2	11.1	4	23.5
	Negative	16	76.2	3	15.0	15	83.3	12	70.6
	Missing	1		2		4		5	
Overall impression of agent	Positive	1	4.8	12	60.0	3	16.7	0	0.0
	Neutral	8	38.1	6	30.0	4	22.2	9	52.9
	Negative	12	57.1	2	10.0	11	61.1	8	47.1
	Missing	1		2		4		5	
Like to use agent in the future	No	13	61.9	4	20.0	10	55.6	8	47.1
	Yes	3	14.3	10	50.0	3	16.7	0	0.0
	Undecided	5	23.8	6	30.0	5	27.8	9	52.9
	Missing	1		2		4		5	

The percentage of subjects experiencing at least one AE related to application of the agents was 86 % (18/21) for P1, 25 % (5/20) for P2, 89 % (16/18) for P3, and 44 % (8/18) with R. The majority of AE were skin reactions and were classified as of mild severity. The number of patients experiencing a related AE of at least moderate severity was 3 (14 %) for P1, 0 for P2, 1 (6 %) for P3, and 0 for R. No serious AE were observed.

## Discussion

Hand disinfection with an alcohol-based hand rub is the most important measure in infection control. Many aspects influencing hand hygiene compliance have been identified, and it is well accepted that skin compatibility plays an important role [8].

This is particularly evident for norovirus infections. These viruses are highly transmissible and hand hygiene compliance is essential to prevent and manage outbreaks. Since non-enveloped viruses are typically highly resistant to disinfectants, special formulations for hand rubs are indicated. Activity against non-enveloped viruses such as noro- and adenovirus is based either on a high concentration of ethanol as active ingredient or supplementation with phosphoric acid. However, these special formulations may impair skin tolerability and impact negatively on healthcare workers’ compliance with hand hygiene.

Because of the paucity of data investigating user acceptance of hand hygiene preparations, the objective of this clinical trial was to evaluate the safety and tolerability of regular use of different virucidal hand rubs within the scope of post-marketing surveillance [9]. Furthermore, to examine to what extent different virucidal compositions (e.g. high ethanol concentration versus phosphoric acid) offer good skin compatibility and user acceptance.

Because there was no knowledge of the differences to be expected regarding the safety and tolerability of the investigational products, a pilot study with a crossover design was planned with a manageable number of subjects that took into consideration potential demographic differences and the possible external influence of time (e.g. influence of cold weather on the skin) but simulating an as near-to-real-life scenario as possible. To objectively characterize skin compatibility, TEWL was assessed as a primary and corneometry as a secondary outcome. Both parameters quantitatively describe the barrier function of the skin and have been well established in investigations and clinical trials on topical preparations and cosmetics [10].

Analysis of TEWL after the intervention period revealed significantly lower TEWL for P2 and P3 as compared to the reference product and P1. These results suggest a better skin barrier function after application of P2 and P3. After application of P3, corneometry was significantly higher compared to the reference product, which again indicates superior skin barrier function. However, this effect was not seen for P2, where corneometry did not differ from P1 or the reference product.

Skin status score was highest after application of P3 and P1. These preparations were also rated worst by the volunteers. However, remarkably, the reference product was not rated very well either. The results indicate poor skin compatibility and impaired user acceptance, especially for those products containing phosphoric acid as supplemental ingredient against non-enveloped viruses.

Since the study was blinded, it was not possible to consider specific application times with respect to virucidal activity. Had the application times tested for virucidal activity specified by the manufacturers been observed, P2 would have had to have been rubbed in twice as long (2 min) as P1 and P3 (1 min each), meaning double the volume would have been applied, which hypothetically might have increased the interactions of P2 with the skin.

From a mechanistic point of view, preparations that are active against non-enveloped viruses are required to damage viral proteins. This leads to a conflict of aims, because damage can also be caused to surface proteins of the skin and result in AEs. The number of AEs was higher than expected for all the preparations tested. Due to the high number of AEs, only 14 out of 22 patients completed all the intervention periods. Most of the AEs were classified as related to agent and of mild severity.

In assessing the large number of AEs, it should be considered that in this investigation application of the hand rubs (90 mL) differed to clinical practice in that it took place within a condensed period of time (90 min). Mechanical effects caused by constant rubbing must therefore also be considered to play an important role in the aetiology of the AEs.

Comparing the results from TEWL and corneometry measurements on the one hand, and the clinical skin score and AEs on the other, the data from this trial appear to be inconsistent. P3, for example, showed low TEWL, suggesting fair skin barrier function but high skin status score and a high number of AEs, which indicates impaired skin compatibility. From this we conclude that the skin barrier function could not be assessed reliably within the trial setting. These parameters might have been influenced by non-volatile compounds contained in the investigational products, especially taking into account that in our study rub application took place within a condensed period. Thus, unlike the clinical setting, where opportunities for hand disinfection are present over the whole shift, and the hands have the chance to dry in between applications, in our trial the test preparations accumulated on the skin. The theory that accumulation of non-volatile compounds constricted reliable TEWL analysis was supported by the fact that the values in P1 and the reference product were comparable, since apart from supplementation of P1 with phosphoric acid the composition of the preparations was identical.

What conclusions can be drawn from these results with regard to optimizing the study design of future trials? Application of the test preparations could take place over a longer period of time. However, extending single interventions puts in question the feasibility of the study. Another option would be to extend the period between intervention end and assessment of the skin barrier.

What conclusions can be drawn from the study with regard to the investigational products? We conclude that all the preparations can be improved with regard to skin compatibility and user acceptance. Evaluation of the skin status score and the AEs suggests that preparations containing phosphoric acid are likely to result in reduced skin compatibility and user acceptance despite their well-documented in-vitro activity against non-enveloped viruses. In summary, this trial shows that there is room for improvement with regard to study design and outcome; it also provides essential information on the skin compatibility of different virucidal hand rubs.

## Abbreviations

P: Product
R: Reference product
TEWL: Transepidermal water loss
CI: Confidence interval
AEs: Adverse events
MedDRA: Medical Dictionary for Regulatory Activities
PP: Per-protocol population

## References

[1] European Centre for Disease Prevention and Control (2012). Surveillance of healthcare-associated infections in Europe, 2007.

[2] Erasmus V, Daha TJ, Brug H (2010). Systematic review of studies on compliance with hand hygiene guidelines in hospital care. Infect Control Hosp Epidemiol.

[3] Dettenkofer M, Block C (2005). Hospital disinfection: efficacy and safety issues. Curr Opin Infect Dis.

[4] Patel MM, Hall AJ, Vinjé J, Parashar UD (2009). Noroviruses: a comprehensive review. J Clin Virol.

[5] Mattner F, Sykora KW, Meissner B, Heim A (2008). An adenovirus type F41 outbreak in a pediatric bone marrow transplant unit: analysis of clinical impact and preventive strategies. Pediatr Infect Dis J.

[6] Fretz R, Schmid D, Jelovcan S (2009). An outbreak of norovirus gastroenteritis in an Austrian hospital, winter 2006–2007. Wien Klin Wochenschr.

[7] Tupker RA, Willis C, Berardesca E (1997). Guidelines on sodium lauryl sulfate (SLS) exposure tests. A report from the Standardization Group of the European Society of Contact Dermatitis. Contact Dermatitis.

[8] Pittet D, Hugonnet S, Harbarth S (2000). Effectiveness of a hospital-wide programme to improve compliance with hand hygiene. Infection control programme.. Lancet.

[9] Kampf G, Löffler H (2010). Hand disinfection in hospitals - benefits and risks. J Dtsch Dermatol Ges.

[10] Pinnagoda J, Tupker RA, Agner T, Serup J (1990). Guidelines for transepidermal loss of water (TEWL) measurement. A report from the Standardization Group of the European Society of Contact Dermatitis. Contact Dermatitis.

